# Dose-related effect of acetylcholine on human gingival blood flow

**DOI:** 10.1186/s12903-024-05169-7

**Published:** 2024-11-17

**Authors:** Tamás László Nagy, Barbara Mikecs, Zsolt M. Lohinai, János Vág

**Affiliations:** Department of Restorative Dentistry and Endodontics, Faculty of Dentistry, Semmelweis University, H-1088 Budapest, Szentkirályi utca 47, Budapest, Hungary

**Keywords:** Acetylcholine, Endothelium-dependent vasodilation, Gingiva, Blood flow, Dose-dependent

## Abstract

**Background:**

This study investigates the dose-response relationship of acetylcholine (ACh) on healthy human gingival blood flow (GBF). Understanding this dose-response relationship contributes to studying vasodilatory mechanisms in various pathological conditions.

**Methods:**

The study involved 22 young healthy men (21 - 32 years) to investigate the dose-response relationship of ACh on GBF. Semi-circular wells were created on the labial surface of the upper right second incisor (FDI #12) and upper left first incisor (FDI #21), including the gingival sulcus, for the application of drugs. ACh-chloride solutions at 0.1, 1, and 10 mg/mL were administered to the gingival sulcus of tooth FDI #12 with a Hamilton syringe. Physiological saline was applied on the contralateral side to FDI #21 as a control. The GBF was measured non-invasively by the laser speckle contrast imaging method in four 1mm high adjacent regions: coronal, midway1, midway2, and apical, and was expressed in a laser speckle perfusion unit (LSPU). After the baseline blood flow recording, ACh doses were applied sequentially, with washout periods in between. Data were statistically analyzed using a linear mixed model.

**Results:**

The GBF did not change on the saline site throughout the experiment. The GBF was significantly higher at the coronal region after all ACh doses (baseline: 218±31 LSPU, and 227±38 LSPU *p *< 0.05, 239±40 LSPU *p *< 0.001, 291±54 LSPU *p *< 0.001, respectively) compared to the saline. It was also elevated following 1 and 10 mg/mL at the midway1 (245±48 LSPU, *p *< 0.05, 293±65 LSPU *p *< 0.001). At midway2 and apical, only the 10 mg/mL dose was effective (285±71 LSPU, *p *< 0.001; 302±82 LSPU, *p *< 0.001).

**Conclusions:**

Our findings suggest a dose-dependent vasodilation to ACh, emphasizing its role in human gingival microcirculation. Only the 10 mg/mL ACh could evoke remote vasodilation 3 mm from the application. The described method could facilitate the investigation of endothelium-dependent vasodilation in disorders affecting microcirculation, such as periodontitis or diabetes.

## Background

Besides post-occlusive reactive hyperemia tests and heat tests, assessing acetylcholine (ACh) induced vasodilation in the skin is a valuable surrogate indicator for detecting endothelial dysfunction-related disorders, including cardiovascular disease (CVD), Raynaud’s phenomenon, and diabetes [1, 2]. However, due to its commonly used intra-arterial application method [3, 4], the ACh test has some drawbacks, i.e., the invasivity. However, more convenient exploratory study methods exist. For instance, another way to apply ACh is to perform iontophoresis on the skin due to its easy accessibility [2, 5, 6], which is less invasive than the intra-arterial route. However, it can cause several side effects, such as erythema, edema, small punctate lesions, painful burns, tingling, and itching [7]. Furthermore, the current or the vehicle of the drug themselves could also cause vasodilation [8]. A novel transsulcular diffusion method was recently developed by our group in the gingiva to study local and regional microcirculatory changes [9–11]. Contrarily to the skin, ACh can penetrate easily through the gingival sulcus, even without iontophoresis [11].

ACh produces endothelium-dependent vasodilation in vessels [12]. ACh initiates a signal cascade after binding its muscarinic receptor on the vascular endothelial cell surface, which activates the medium- and low-conductance Ca^2+^-activated K^+^-channels (IK_Ca_ and SK_Ca_) [13] located in the plasma membrane, producing hyperpolarization [14]. The consequently increasing intracellular Ca^2+^-concentration activates endothelial nitric oxide synthase (eNOS), producing nitric oxide (NO), which rapidly diffuses into the surrounding vascular smooth muscle cells [15] where the intracellular Ca^2+^-concentration decreases due to the activation of Ca^2+^-dependent K^+^-channels [16] and Ca^2+^-pumps located in the sarcolemma and the sarcoplasmic reticulum membrane [17]. This mechanism results in the relaxation of the vascular smooth muscle cells. However, in the case of periodontitis, the bioavailability of the NO pathway is decreased as a sign of endothelial dysfunction [18].

A growing number of evidence indicates an interplay between periodontitis and systemic conditions characterized by microcirculatory insufficiencies, such as CVD [19] and diabetes [20]. In advanced periodontitis, the flow-mediated dilation in the brachial artery induced by cuff occlusion is severed compared to the healthy control, contrary to the nitroglycerin-induced one [21]. Intensive treatment of severe periodontitis significantly improved the flow-mediated dilation of the brachial artery, but no difference was observed for nitroglycerin-induced dilation between periodontitis and healthy patients [22]. The periodontal condition can influence the cardiovascular status, but on the other hand, the systemic condition could affect the periodontal microcirculation as well. Animal experiments indicated attenuation in vascular reactivity of the gingiva in experimentally induced type 2 diabetes [23]. Aortic vascular function and oral microcirculation were also affected in an animal model of stroke and periodontitis [24]. In patients with type 2 diabetes or gestational diabetes, higher periodontal capillary density was found compared to healthy individuals [25, 26]. Additionally, periodontitis could induce local changes in microvascular function in the gingiva [27, 28].

The ACh test could be a valuable method for assessing vascular reactivity in gingival microcirculation and endothelial function in various pathological conditions. However, the proper doses need to be established to maximize the statistical power and limit the possible side effects. Additionally, more ACh might exert vasoconstriction directly on the smooth muscle cells instead of vasodilation. For example, vasodilation is observed in coronary circulation after intra-arterial administration of low-dose ACh but vasoconstriction after high doses [3, 4].

Previously, 10 mg/mL ACh applied to the sulcus was effective in vasodilation in the human gingiva not just at the application (coronal region) but even 3 mm apically from the application site [11]. The effect of ACh was gradually decreased from coronally to apically. It was assumed that the apical vasodilation is partially due to flow-mediated dilation [9] rather than the transportation of ACh, as the arterial blood flow direction is from apically to the coronally [29, 30].

This study aimed to determine the lowest concentration of transsulculary applied ACh, which could evoke significant apical vasodilation. The null hypothesis was that a concentration between 0.1 mg/mL and 10 mg/mL would not elicit remote vasodilation in gingiva. The secondary null hypothesis was that none of the investigated concentrations would elicit vasodilation in any region.

## Methods

### Study population

In the previous experiment [11], 10 mg/mL ACh was applied to the sulcus in 12 subjects. Based on the result (effect size), the sample size required to detect the interaction effect of site (control vs. test) and period (baseline vs. ACh) at the apical region was *n* = 6, calculated with a power of 0.95 and assuming a correlation of *r* = 0.5 between repeated measurements. However, the sample size was increased in this experiment to 20 to detect more minor changes due to the reduced concentration. In the current experiment, a 10% drop-out was calculated due to the failure in the measurement. Therefore, 22 volunteers were recruited in this study (registry number at ClinicalTrials.gov is NCT06454084, registration date: 01.09.2023.). To eliminate the potential influence of the menstrual cycle, a factor previously shown to impact the microcirculation of oral tissues [31], the effect of ACh on GBF was assessed in males.

Only young subjects between 21 and 32 years were selected to avoid the effects of aging [5]. The study was carried out following the Declaration of Helsinki. Ethical approval was granted by the Hungarian National Public Health and Medical Officer Service (57734-7/2022/EÜIG) based on the opinion of the Ethics Review Committee of the Medical Research Council (ETT-TUKEB). Each subject received written information about any possible risk and details of the measurement. Signed informed consent was obtained.

The young male volunteers were selected based on their excellent general and oral health and having at least 5 mm keratinized gingiva at the labial side of the upper front teeth. Exclusion criteria were smoking, medication, caries, periodontitis, or restoration with insufficient marginal integrity in the upper front region. In addition, the participants were told not to brush their teeth or eat and drink 60 min before the measurement to avoid any extrinsic and intrinsic stimulus, which could affect the GBF.

### Site preparation for GBF measurement

The patients were seated in a dental chair in a standardized supine position. They could adapt to the calm conditions in the quiet room, which had an average temperature of 26 °C for 15 min. Systolic and diastolic blood pressure and heart rate were measured with an automatic blood pressure monitor (Omron M2, Omron Healthcare Inc., Kyoto, Japan) on the left arm three times during the session: right after the patient’s arrival, immediately before, and at the end of the GBF measurement.

The measurement report recorded the patient’s name, date of birth, gender, operator’s name, and ambient temperature during the resting period.

**Fig. 1:**
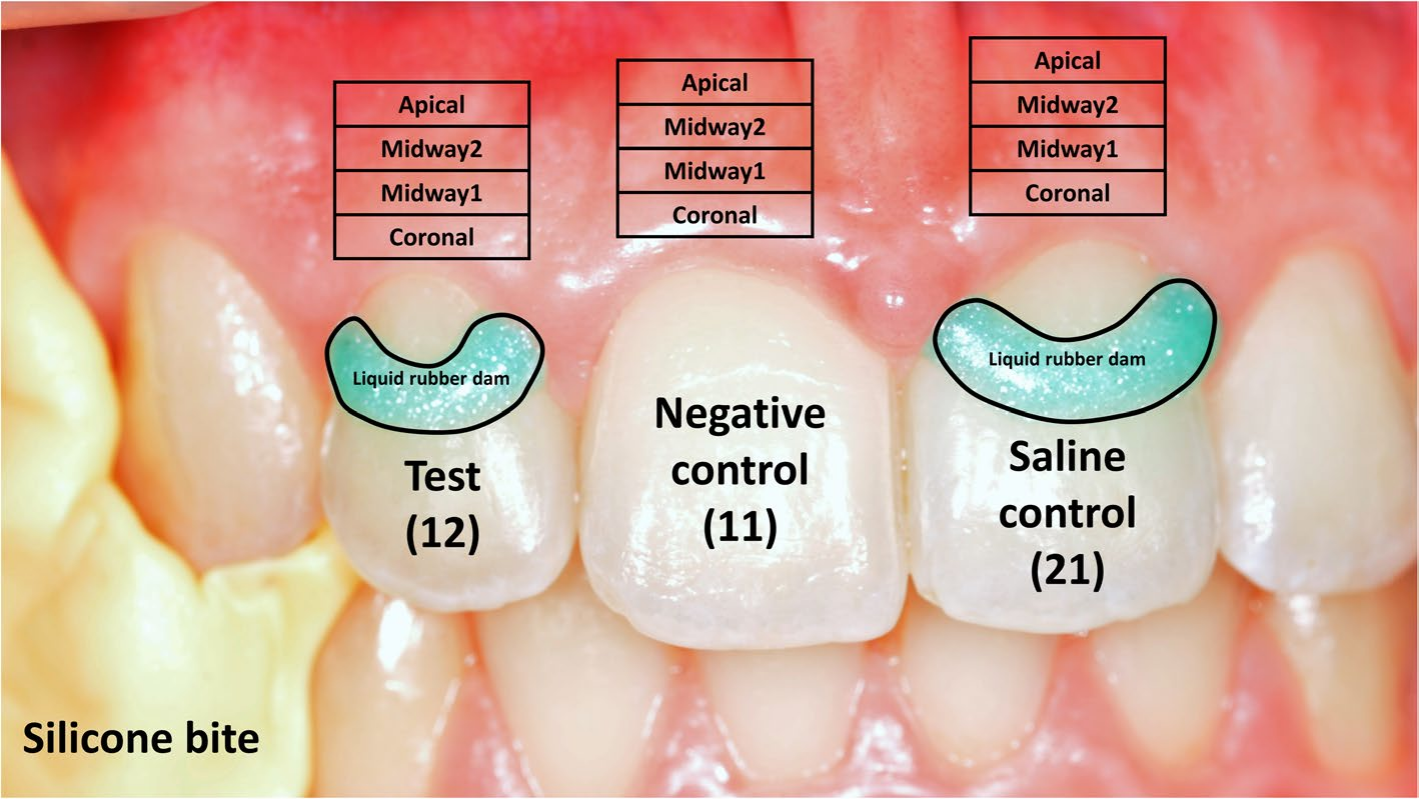
Site preparation for the gingival blood flow measurement. The FDI #12 tooth was the test site, and the FDI #21 was the physiological saline control site. The solutions in the gingival sulcus were kept in place by a liquid rubber dam (green). During the measurement, a silicone bite (yellow, left side) fixed the jaw. The FDI #11 tooth was a negative control, so no preparation was applied. Four regions of interest were set with 1 mm height and 5 mm width above the investigated teeth (coronal, midway1, midway2, and apical)

The patient’s head was fixed by a vacuum pillow (Spandex^®^, Hager&Werken, Germany), their lips were retracted carefully with a lip retractor (OptraGate, Ivoclar Vivadent AG, Liechtenstein), and their jaw was stabilized using a silicone bite. These actions were essential to minimize any movements. Two semi-circular wells were created on the labial surface of the upper right second incisor (FDI#12, test site) and another of the upper left first incisor (FDI#21, saline control site) with a light-cured resin-based barrier material (OpalDam Green, Ultradent Products Inc., USA). No well was formed at the upper right first incisor. Therefore, it was used as a negative control (negative control site) (Fig. 1.).

### GBF measurement

After finishing all the preparatory settings, the baseline GBF measurement started. The GBF was measured by a camera using a laser speckle contrast imaging method (LSCI, PeriCam PSI HR System, Perimed AB, Stockholm, Sweden). The GBF was recorded in an arbitrary laser speckle perfusion unit (LSPU) by the Pimsoft Software (PeriCam PSI HR, Perimed AB). The LSCI illuminates the object with infrared laser light (785 nm). The laser light reflects from static objects and forms a speckle pattern. However, moving cells blur this pattern proportionally to their velocity. By calculating the contrast of the captured image, LSCI can assess the microcirculation in a non-contact and non-invasive manner with high spatial and temporal resolution. Adapting this method to the human gingiva revealed good reproducibility and repeatability [32]. The measurement area was 2 × 3 cm as the maximum achievable at the given temporal (10 images per second) and spatial resolution (0.05 mm/pixel). In addition, the angle between the measured surface and the camera increases by the horizontal distance, lowering the reliability of the distance measurement, which requires region selection. Therefore, tooth 21 was chosen for reference measurement as tooth 22 would not fit into the field of view.

### Application of drugs

Acetylcholine chloride was diluted in sterile physiological saline in an Eppendorf vial. In our previous study on the human gingiva [11], the 10 mg/mL ACh was effective. Therefore, besides the 10 mg/mL solution, two additional concentrations, 0.1 and 1 mg/mL, were used by diluting the stock. Hamilton syringes were applied to the wells, and an endodontic aspirator was used to remove the solution. The ACh-solutions were shaken with a Vortex mixer before aspirating by a Hamilton syringe (Model 75 RN SYR, Hamilton, Switzerland). The three syringes filled with ACh-solutions and two more syringes filled with pure physiological saline were then put into a block heater (Dry Block Thermostat DBI-100, Boeckel GmbH, Hamburg, Germany) to keep them at 37 °C before their application. Every syringe was filled with 3 µL solution.

**Fig. 2:**
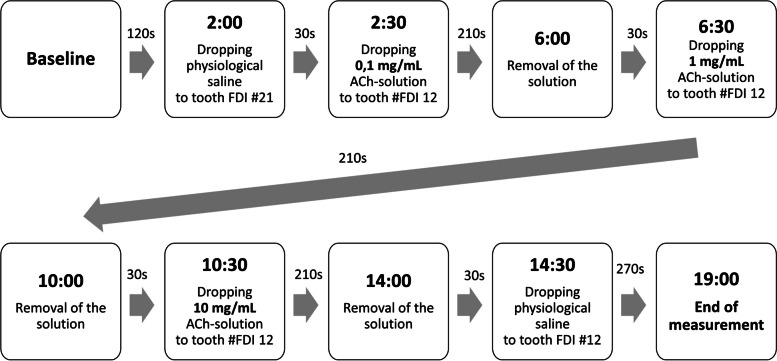
Order of the application and removal of the different concentrations of ACh-solutions. Baseline gingival blood flow was measured for 90 s, and at 2:00, physiological saline was dropped on the FDI #21 tooth. The three ACh-solutions were applied to the gingival sulcus of FDI #12 tooth at 2:30 (0,1 mg/mL), at 6:30 (1 mg/mL), and at 10:30 (10 mg/mL). Every solution was kept in the gingival sulcus for 210 s and was removed by an endodontic aspirator (at 6:00, 10:00, and 14:00, respectively). The following solution was applied 30 s after removing the previous solution. Physiological saline was dropped into the gingival sulcus of FDI #12 tooth at 14:30 to remove the effects of the final ACh dose

The timing of application and removal of the solutions is shown in Fig. 2. Baseline GBF was recorded for 120 s after the start of the measurement. During the next 30 s, 3 µL of physiological saline solution was dropped to the gingival sulcus of the upper left first incisor (saline control site) and left there throughout the measurement. For the next 30 s, 3 µL of 0.1 mg/mL ACh solution was applied to the gingival sulcus of the maxillary right second incisor (test site) and left undisturbed in the sulcus for 210 s. The solution was then removed. For the next 30 s, 3 µL of 1 mg/mL ACh-solution was applied to the gingival sulcus and removed after 210 s. For the next 30 s, 3 µL of 10 mg/mL ACh was applied to the gingival sulcus and removed after 210 s. Finally, physiological saline was applied to the test site for 270 s to wash out the ACh. A saline washout between each ACh solution was not performed, as most of the ACh was absorbed by the tissue, as indicated by the vasodilation response. In addition, the consecutive concentration was ten times more, minimizing the crossover effect. In contrast, removing the previous ACh solution was necessary, as the solution filled up the space of the wells, preventing the addition of the next solution.

The LSCI measurement was made without interruption throughout the experiment by the temporal resolution of 10 images per second. The Pimsoft Software averaged every ten images during recording. Further averaging of the last 10 s of the periods of interest was applied in MS Excel after exporting the recordings from Pimsoft. The periods were as follows. The 30, 60, and 90 s of baseline, 30 s of physiological saline application to the control site, 30 s of 0.1 mg/mL ACh application to the test site, 60, 120, and 240 s after the application, 30 s of 1 mg/mL ACh application, 60, 120, and 240 s after the application, 30 s of 10 mg/mL ACh application, 60, 120, and 240 s after the application, 60 s of removal of ACh and application saline, and 60, 120 and 240 s after it.

Lastly, the periodontal probing depth and keratinized gingival width were measured with a periodontal probe (UNC 15, Hu-Friedy, Chicago, IL, US). The same investigator conducted the drug application and measuring procedures for all subjects.

### Data analysis

Four regions of interest (ROI) were defined in the Pimsoft Software by the ROI tool above each investigated tooth (Fig. 1) on the keratinized gingiva. Each rectangle-shaped ROI was 1 mm high and 5 mm wide: the coronal, the midway1, the midway2, and the apical ROIs.

### Statistics

The LSPU values are shown in mean ± standard deviation (SD) form in the table and the text. In the figures, the mean and 95% confidence intervals are depicted. The change in LSPU values due to the ACh application was evaluated by comparing the LSPU values measured at the test site with the LPSU values measured at the control site. Using the control site as the reference increases the reliability of the measurement due to control of the subtle variation in the oral cavity conditions (temperature, wetness) and systemic change (for example blood pressure). Furthermore, dropping any fluid might exert a mechanical stimulus affecting the blood flow. The saline application controlled this possible effect.

The last period (210 s) of the application of each ACh concentration was evaluated for statistics. The statistically significant differences were estimated by a linear mixed model with a fixed effect of time, site, and region and a random effect of time and subject. The model also included the baseline values (30, 60, and 90 s periods) as a covariant to adjust the change by time with the difference in baseline values between sites. *P* < 0.05 was accepted as a significant difference after sequential Bonferroni correction. Analyses were made in SPSS (version 28, IBM, USA).

## Results

**Fig. 3:**
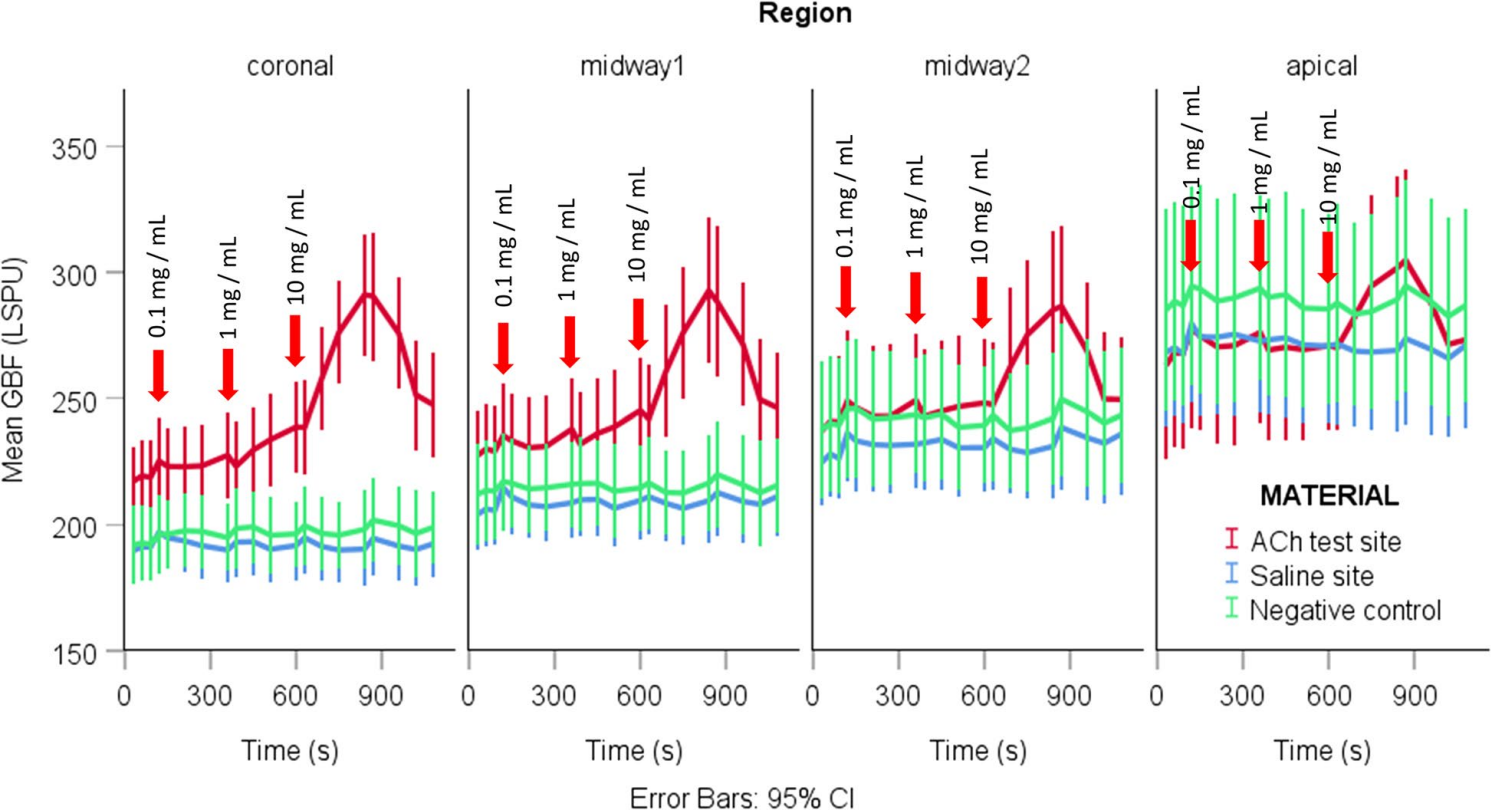
The mean with a 95% confidence interval (error bars) of the gingival blood flow change values concerning the time in the four regions during the measurement. The red curve represents the test site (ACh, FDI #12 tooth), where ACh was applied, the blue curve is for the physiological saline site (Saline, FDI #21 tooth), the green one is for the negative control site (NONE, FDI #11 tooth). The beginning of the ACh administration is depicted by arrows

Table 1 shows the mean GBF in the baseline period and the GBF after removing the 0.1, 1, and 10 mg/mL ACh. Figure 3 depicts the mean GBF changes by time in all four regions during the entire measurement.

**Table 1:** Gingival blood flow before and after various doses of ACh compared to the physiological saline site at the different investigated regions

ROI	Drug	baseline * (LSPU)		0.1 mg/mL (LSPU)		1 mg/mL (LSPU)		10 mg/mL (LSPU)		% change to baseline after 10 mg/mL (%)	
		Mean	SD	Mean	SD	Mean	SD	Mean	SD	Mean	SD
Coronal	ACh	218*	31	227*	38	239***	40	291***	54	33,7	21,3
	Negative control	192	33	195	30	196	29	198	36	3,1	8,4
	Saline	191	28	190	29	192	31	190	33	-0,1	10,9
Midway1	ACh	229	40	238	45	245*	48	293***	65	28,1	19,7
	Negative control	213	44	216	38	215	38	216	43	2,2	9,4
	Saline	205	32	209	32	210	35	209	38	1,9	10,0
Midway2	ACh	240	58	249	60	248	57	285***	71	19,9	16,5
	Negative control	239	60	243	51	239	52	242	59	1,8	9,6
	Saline	226	37	232	39	231	39	231	46	1,9	10,1
Apical	ACh	266	84	276	81	271	74	302***	82	15,3	15,9
	Negative control	287	89	294	82	285	85	289	91	1,0	9,3
	Saline	269	63	273	63	271	69	269	72	-0,2	9,0

*SD *Standard Deviation, *ACh *acetylcholine, *Saline *physiological saline control

* indicates a significant difference between the sites, *p < 0,05, **p < 0,01, ***p < 0,001

**Fig. 4:**
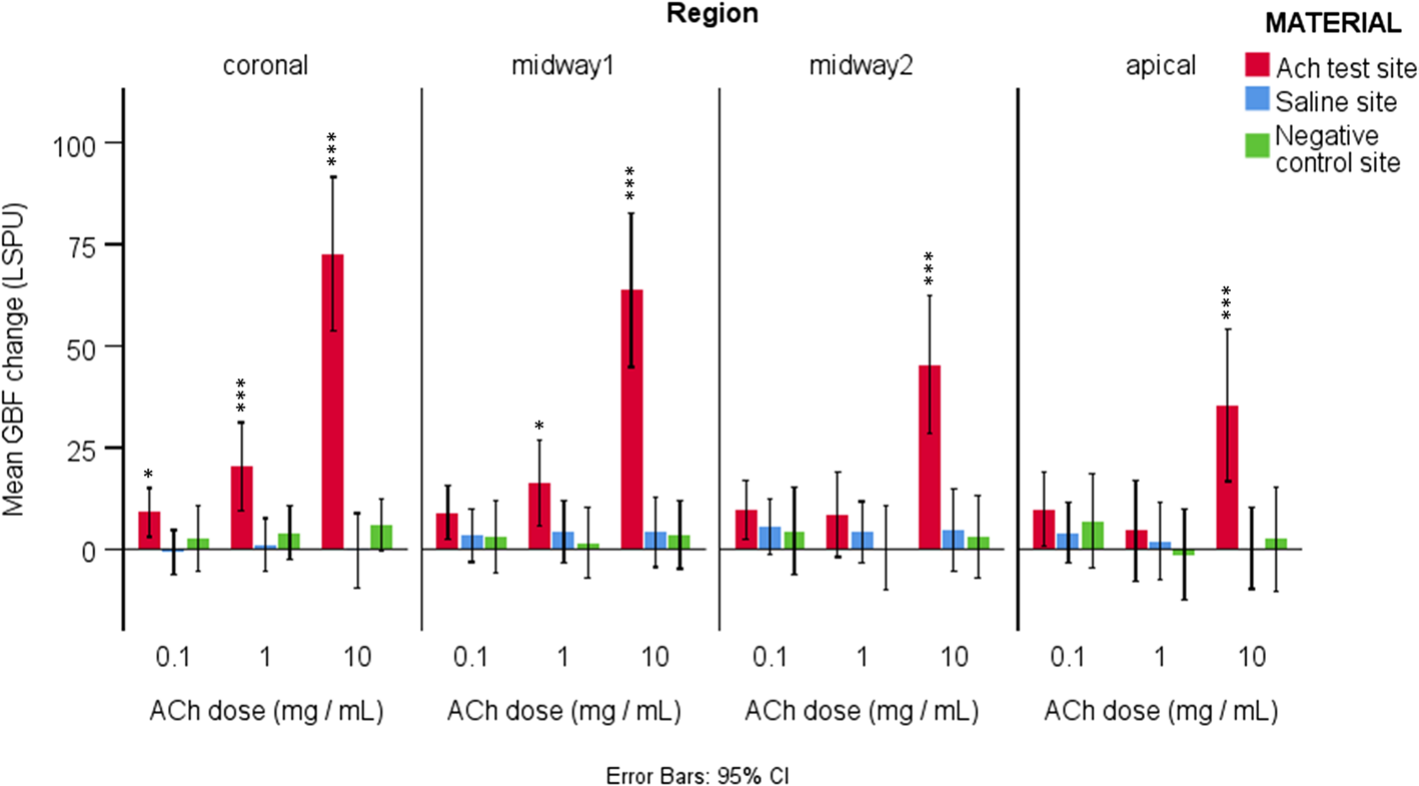
The mean GBF change (= post-application GBF – baseline GBF) expressed in LSPU with a 95% confidence interval (error bars) concerning time in all four investigated regions 210 s after ACh was applied in 0.1, 1, and 10 mg/mL doses. Gingival blood flow was normalized to baseline blood flow. The vasodilatory effect induced by ACh decreased with increasing distance from the application site (from coronal to apical). The higher the concentration of ACh in the applied solution (e.g., 0,1 mg/mL vs. 10), the more distant it could enhance a significant change in blood flow. The gingival blood flow values are expressed in LSPU, * indicates a significant difference between the examined effect of the solutions, *p < 0.05, **p < 0.01, ***p < 0.001

The time x agonist x region interaction was significant. Therefore, the difference between sites was evaluated separately for each region and agonist. (Fig. 4).

After 0.1 mg/mL ACh-solution, the elevation in GBF was significantly higher in the coronal region of the test site than in the saline site after adjusting the mean baseline blood flow (9.04 ± 13.59 vs. -0.73 ± 12.28, *p* < 0.05) (Fig. 4). In the midway1, midway2, and apical regions; however, the test site did not significantly differ from the saline site (*p* = 0.279, *p* = 0.743, *p* = 0.873). No significant differences were observed between saline and the negative control sites in either region (*p* = 0.519, *p* = 0.967, *p* = 0.965, *p* = 0.873).

After 1 mg/mL ACh, the increase in GBF was significantly higher in the coronal (20.23 ± 24.55 vs. 1.04 ± 14.88, *p* < 0.001) and midway1 (16.26 ± 23.65 vs. 4.31 ± 17.12, *p* < 0.05) regions of the test site than in the saline site (Fig. 4). In the midway2 and apical regions, the test site was not significantly different from the saline site (*p* = 0.687, *p* = 1.000). No significant differences were observed between saline and the negative control sites in either region (*p* = 0.557, *p* = 0.672, *p* = 0.687, *p* = 1.000).

After 10 mg/mL ACh, the increase in GBF was significantly higher in the coronal (72.59 ± 42.78 vs. -0.37 ± 20.64, *p* < 0.001), midway1 (63.76 ± 42.71 vs. 4.20 ± 19.38,*p* < 0.001), midway2 (45.40 ± 38.40 vs. 4.68 ± 22.86, *p* < 0.001), and apical (35.35 ± 42.19 vs. 0.31 ± 22.74, *p* < 0.001) region on the test site compared to the saline site (Fig. 4). No significant differences were observed between saline and the negative control sites in either region (*p* = 0.234, *p* = 0.978, *p* = 0.878, *p* = 0.599).

## Discussion

The first null hypothesis was partially rejected, as the highest concentration of ACh induced significant gingival hyperemia at the apical region. The second null hypothesis was also partly rejected, as even the smallest concentration induced significant vasodilation in the coronal region but not in the other three regions.

Our results can be compared with previous studies on the skin but with some restrictions. Several studies examined the dose-response effects of ACh on the skin vasculature under healthy and pathological circumstances [2, 5, 6, 33]. These investigations included both sexes and a wide range of ages. Moreover, the focus was mainly on the vasodilatory capacity and the microvascular responses. Additionally, the measurements were carried out primarily on the skin because it is an easily accessible tissue.

Rossi et al. [5] evaluated the endothelial function and intrinsic vasodilatory capacity of skin microvessels in healthy elderly and young humans with laser Doppler flowmetry: 15 elderly (10 males and 5 females, 66–88 years) and 15 young control (8 males and 7 females, 23–49 years) were involved, into whom 10 mg/mL ACh-solutions were delivered by iontophoresis. When the plateau phase was established, the drug administration was repeated eight times, resulting in a total accumulation of 41.48 µg (6 times 3.77 µg, then once 7.54 µg, and finally 11.32 µg). The estimated absorbed amount was calculated based on the iontophoresis theory paper of Moor Instruments [34]. Each dose was applied for 20 s, followed by a 40 s period before the subsequent iontophoresis activation. Therefore, the total adequate time was 160 s. The skin blood flow increased with every additional dose from 20 to 100% with the highest one. Conversely, our study found a 33% increase in coronal GBF after 210 s application of 10 mg/mL ACh. The absorbed total dose can be compared with the skin only with caution, as no iontophoresis was used in the gingiva. Our data suggest that ACh can penetrate through the gingival sulcus more quickly than in the skin due to the high natural permeability of the junctional epithelium of the gingival sulcus [35], where the permeability is the highest for physiological reasons among all oral epithelial segments [36].

Christen et al. [37] examined the vasodilatory capacity of the skin microcirculation in 26 healthy young men after incremental ACh concentrations using transdermal iontophoresis with six different current charges and laser Doppler imaging. With 10 mg/mL ACh in a charge-dependent manner, the pattern of reaching the plateau phase was similar to this study and previous results in gingiva [11]. However, the blood flow showed a maximal 7.1-fold increase compared to the baseline [37], but we experienced only a 1.3-fold increase compared to the baseline. The laser Doppler imaging measures microcirculation differently than the LSCI [38], which might be another reason for the difference in the results.

De Matheus et al. [2] registered the cutaneous microvascular responses of 30 healthy patients (12 males and 18 females, 21–31 years) using iontophoresis with LSCI. Six concentrations of 20 mg/mL ACh-solution were applied from 0.7 µg to 3.4 µg with a total amount of 11.9 µg. The blood flow increased by 61% [2], matching our results (33% increase with 10 mg/mL).

Iontophoretic stimuli have the potential to activate multiple pathways that influence vasomotor reactions [8]. Consequently, distinguishing whether the vasomotor changes are attributed to the electrical stimulus or the vasoactive agent becomes problematic [39]. This ambiguity holds significance as it complicates the standardization of vasodilation induction methods [8, 38]. Thus, a more precise approach is favored, such as directly applying ACh near microcirculatory units present at the gingival sulcus. However, this method is not practical for the skin due to its impermeability.

Stewart et al. [6] conducted a study involving 12 healthy participants (8 males and 4 females, aged 17–25 years) utilizing intradermal microdialysis instead of iontophoresis with laser Doppler flowmetry. They administered five concentrations of ACh solution: 0.0018 mg/mL (0.073 µg), 0.018 mg/mL (0.73 µg), 0.18 mg/mL (7.3 µg), 1.8 mg/mL (73 µg), and 18 mg/mL (727 µg), totaling 808 µg of the applied agent. The blood flow exhibited a direct increase corresponding to the dose, ranging from 10 to 80%. In contrast, only a 33% increment in coronal gingival blood flow was observed at our highest 10 mg/mL concentration. This relatively smaller effect could be attributed either to the diffusion capacity of the sulcus epithelium or to a lower ACh receptor density in this region compared to the skin.

Our findings indicate that the externally administered ACh evokes significant gingival vasodilation, in line with the findings of others [11, 40, 41]. Intravenous ACh increases the blood flow of rat gingiva in a site-specific manner; it is more involved in the interdental papilla than in the other parts of the gingiva [41]. Intracarotid ACh injection significantly increased the ipsilateral gingival blood flow in anesthetized dogs [40]. It increases GBF through vasodilation by activating endothelial cells to release NO [42]. Transsulcular ACh application elicits quick, upstream (toward the alveolar mucosa), spreading vasodilation mediated at least partially by NO [9, 15]; thus, ACh is a surrogate marker for investigating endothelium-dependent vasodilation [2]. Interestingly, ACh-induced vasodilation in the gingiva is more pronounced (higher and faster) in men than in women, contrary to the direct effect of the NO donor [11]. This higher endothelium-dependent vasodilation in men could be a contributing factor to the greater severity of periodontal disease observed in men compared to women [43].

Despite the evidence that externally added ACh elicits gingival vasodilation, the literature remains unclear on how ACh is released in the gingival vascular bed and its intrinsic mechanism of action in a physiological environment. Theoretically, it may be released from the innervating vasomotor nerves. Still, muscarinic cholinoceptor antagonist atropine did not affect the gingival vasodilatation elicited by electrical stimulation of the inferior alveolar, facial, or glossopharyngeal nerves in anesthetized cats [44]. Contrarily, electrical stimulation of the central cut end of the lingual nerve in anesthetized rats, which reflexively activates the parasympathetic vasodilator fibers innervating the gingiva, was recently reported to have a cholinergic mechanism [41]. However, the functional implication of this trigeminal-parasympathetic reflex vasodilation remains unclear. Other possible ACh sources, like the bloodstream, are unlikely, as ACh is rapidly broken down by acetylcholinesterase [45].

In patients with periodontal disease, ACh levels are significantly elevated in saliva and gingival crevicular fluid, whereas gingival crevicular fluid levels of butyrylcholinesterase, a serine hydrolase that catalyzes the hydrolysis of esters of choline, including ACh [46], are significantly decreased [47]. Therefore, non-neuronal cholinergic mechanisms may also influence the etiopathogenesis of periodontal inflammation [47]. Furthermore, periodontitis exhibited impaired endothelial-dependent local and systemic vasorelaxation responses to ACh [48].

## Conclusion

Unlike lower concentrations, the 10 mg/mL ACh concentration demonstrated potency, enabling a remote effect of up to 3 mm away from the application. LSCI combined with local administration of the ACh is a promising, non-invasive, simple approach for assessing endothelial function in human gingiva. Consequently, this suggested method holds potential for studying endothelial dysfunction in vivo, as impairment in spreading vasodilation is a marker for pathologic vascular conditions.

## Data Availability

Data is provided within the manuscript.

## Abbreviations

ACh: acetylcholine
GBF: gingival blood flow
LSCI: laser speckle contrast imaging
LSPU: laser speckle perfusion unit
ROI: region of interest
